# Genomic diversity of SARS-CoV-2 during early introduction into the Baltimore–Washington metropolitan area

**DOI:** 10.1172/jci.insight.144350

**Published:** 2021-03-22

**Authors:** Peter M. Thielen, Shirlee Wohl, Thomas Mehoke, Srividya Ramakrishnan, Melanie Kirsche, Oluwaseun Falade-Nwulia, Nídia S. Trovão, Amanda Ernlund, Craig Howser, Norah Sadowski, C. Paul Morris, Mark Hopkins, Matthew Schwartz, Yunfan Fan, Victoria Gniazdowski, Justin Lessler, Lauren Sauer, Michael C. Schatz, Jared D. Evans, Stuart C. Ray, Winston Timp, Heba H. Mostafa

**Affiliations:** 1 Johns Hopkins University Applied Physics Laboratory, Laurel, Maryland, USA.; 2 Johns Hopkins Bloomberg School of Public Health, Baltimore, Maryland, USA.; 3 Department of Computer Science, Johns Hopkins University, Baltimore, Maryland, USA.; 4 Department of Medicine, Division of Infectious Disease, Johns Hopkins University School of Medicine, Baltimore, Maryland, USA.; 5 NIH, Fogarty International Center, Bethesda, Maryland, USA.; 6 Department of Emergency Medicine, Johns Hopkins University, Baltimore, Maryland, USA.; 7 Department of Pathology, Division of Medical Microbiology, Johns Hopkins University School of Medicine, Baltimore, Maryland, USA.; 8 Departments of Biomedical Engineering and Molecular Biology and Genetics, Johns Hopkins University, Baltimore, Maryland, USA.

**Keywords:** COVID-19, Genetic variation

## Abstract

The early COVID-19 pandemic was characterized by rapid global spread. In Maryland and Washington, DC, United States, more than 2500 cases were reported within 3 weeks of the first COVID-19 detection in March 2020. We aimed to use genomic sequencing to understand the initial spread of SARS-CoV-2 — the virus that causes COVID-19 — in the region. We analyzed 620 samples collected from the Johns Hopkins Health System during March 11–31, 2020, comprising 28.6% of the total cases in Maryland and Washington, DC. From these samples, we generated 114 complete viral genomes. Analysis of these genomes alongside a subsampling of over 1000 previously published sequences showed that the diversity in this region rivaled global SARS-CoV-2 genetic diversity at that time and that the sequences belong to all of the major globally circulating lineages, suggesting multiple introductions into the region. We also analyzed these regional SARS-CoV-2 genomes alongside detailed clinical metadata and found that clinically severe cases had viral genomes belonging to all major viral lineages. We conclude that efforts to control local spread of the virus were likely confounded by the number of introductions into the region early in the epidemic and the interconnectedness of the region as a whole.

## Introduction

SARS-CoV-2, the virus that causes COVID-19, established itself worldwide within 2 months of its emergence in Wuhan, China (1). As of December 23, 2020, over 78 million confirmed COVID-19 cases and 1.7 million deaths have been reported (2). The enormous health and economic impacts of this virus have led to considerable interest in understanding its origin, spread, and evolution. Generation and analysis of pathogen genomic data have been key components of this research (3–8) and provide critical insights into not only the emergence and spread of SARS-CoV-2 but also the dynamics of emerging infections in general.

In the United States, diagnostic capacity for SARS-CoV-2 was limited until early March 2020 due to regulatory challenges associated with limited Emergency Use Authorization (EUA) for laboratory-developed testing. Retrospective analyses of patient samples using genomic and serological methods now suggests that community transmission was occurring in major US cities as early as late January or early February 2020 (9–11). Ongoing work has continued to deepen our understanding of SARS-CoV-2, including use of pathogen sequence data to help reconstruct the transmission of the virus into and around the United States (12).

Coronaviruses, including SARS-CoV-2, possess proofreading activity that limits genetic variability (13). This genome replication feature, combined with rapid spread and limited immunity during the early phase of the pandemic, has likely limited evolutionary pressure and contributed to the limited genetic diversity observed in SARS-CoV-2 sequences. Efforts to describe this diversity during the early phase of the pandemic have resulted in multiple clade (or lineage) designation systems including those used by the Global Information Sharing of All Influenza Data (GISAID), the NextStrain platform, and COVID-19 Genomics UK consortium (14–16). These approaches are intended to be dynamic and are updated as new diversity is observed in the global sequence data. For example, NextStrain clade designations separate viruses that originated in China in 2019 (Clade 19) from those that were later introduced into Europe in early 2020 (Clade 20) (15). Similarly, the Pango nomenclature system provides a dynamic nomenclature system that is updated based on newly observed viral lineages (14). Importantly, SARS-CoV-2 clade designations are only intended to identify subgroups of virus sequences that share common genetic features, and further in vitro or clinical characterization is required to identify functional differences between clades.

Using sequence data to investigate relationships between virus genetics and patient clinical outcome often relies on specimen repositories or agreements with sample collection facilities that provide limited access to patient demographic and clinical information. Limited access is due to both logistical challenges in obtaining this data and ethical concerns around patient privacy (17). Therefore, although research efforts have produced copious amounts of valuable genetic data and insights into viral circulation (12, 18), studies linking pathogen genetics to demographics, disease severity, and other clinical outcomes are less frequent. By leveraging existing internal networks and established research protocols, research groups within large health systems can fill this gap by rapidly creating and analyzing data sets that link pathogen genomics with clinical and demographic outcomes.

To gain insight into regional viral spread and potential associations between pathogen genetics and clinical outcomes, we performed whole genome sequencing of the SARS-CoV-2 virus from clinical samples in the Johns Hopkins Health System (JHHS), which is centered in Baltimore, Maryland, and spans the entire Baltimore–Washington metropolitan area. For this work, we primarily used the Oxford Nanopore sequencing platform, which has been increasingly used during early outbreak investigations to understand emerging pathogens (19, 20). This portable platform allowed us to begin sequencing rapidly during an ongoing pandemic. To establish SARS-CoV-2 sequencing capacity within the JHHS, we validated and improved upon widely used bioinformatic pipelines for identifying single nucleotide polymorphisms from Oxford Nanopore sequencing data, making use of laboratory controls and sequence validation on the Illumina platform. Here, we explore the relationship between local sequences and those from the broader national and global epidemic and look for possible associations between clade structure and clinical outcomes.

## Results

### Characteristics of SARS-CoV-2 identification in the region.

**Figure 1:**
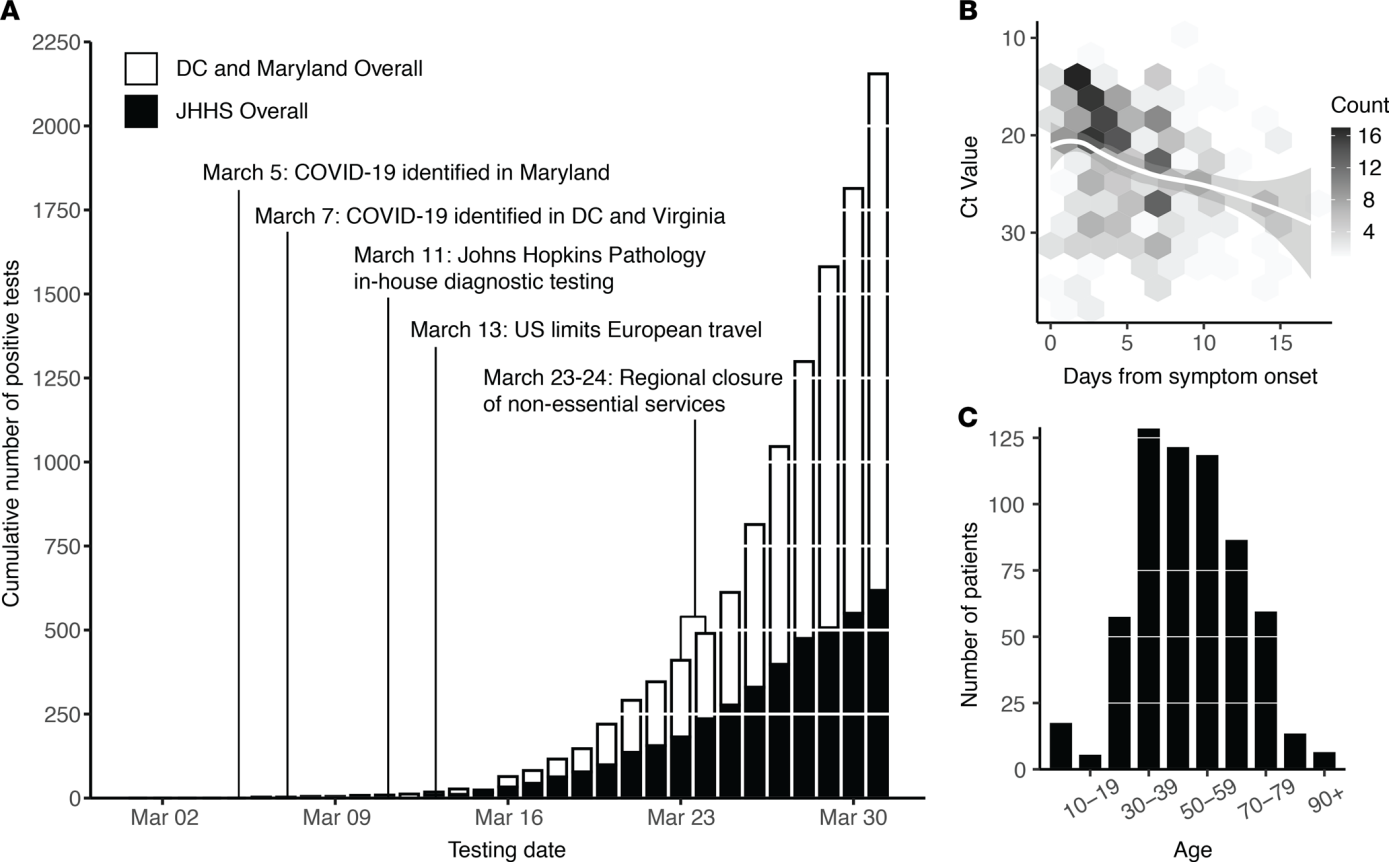
COVID-19 diagnostic response during initial SARS-CoV-2 surveillance in the JHHS. (**A**) Cumulative number of positive tests in Washington, DC, and the state of Maryland (white bars) and within the JHHS (black bars). (**B**) SARS-CoV-2 RT-PCR C_T_ value (S-gene) versus days from patient symptom onset. Data fit with LOESS curve (white regression line). Two outliers (days from onset = 5 weeks, C_T_ value = 30 and days from onset = 28 days, C_T_ value = 31) are not shown. (**C**) Age distribution of SARS-CoV-2 patients within the JHHS. JHHS, Johns Hopkins Health System; C_T_, threshold cycle.

The Baltimore–Washington metropolitan area spans Maryland, Washington, DC, and Northern Virginia, and is an area of high domestic and international transit as well as geopolitical importance. The JHHS is spread throughout the region, including 39 hospitals and clinics throughout Maryland and Washington, DC, with a patient population that also includes residents of Northern Virginia. The first detection of SARS-CoV-2 in the region was reported on March 5, 2020, by the Maryland Department of Health, and a State of Emergency was immediately declared in Maryland (21). Two days later cases were reported by the public health departments in Virginia and Washington, DC. On March 23rd, the Maryland state government instituted regional closure of nonessential businesses, and a stay-at-home order followed on March 30th (Figure 1A).

Molecular diagnosis of SARS-CoV-2 at the JHH Medical Microbiology laboratory began March 11th, using the RealStar SARS-CoV-2 RT-PCR kit from Altona Diagnostics, which was granted FDA EUA after analytical validation (22). The Altona test targets both lineage B betacoronavirus E genes and the SARS-CoV-2 S gene. In the last week of March, the laboratory also began the use of the Cepheid Xpert Xpress SARS-CoV-2, an assay for which the FDA has granted an EUA and that targets the E and N2 genes. The laboratory evaluated a total of 5913 nasopharyngeal swabs and confirmed 620 COVID-19–positive patients (10.4% overall positivity rate) (Supplemental Table 1; supplemental material available online with this article; https://doi.org/10.1172/jci.insight.144350DS1). In total, 603 positive diagnoses were made with the Altona assay in this time period, and 17 positive diagnoses were made with the Cepheid Xpert Xpress SARS-CoV-2 assay. This represented 28.6% of the 2155 confirmed cases in Maryland and Washington, DC, during this period (Figure 1A).

We found that the cycle threshold (C_T_) value of SARS-CoV-2 diagnostic testing performed using the Altona assay was weakly associated with self-reported days from symptom onset (Spearman’s correlation; *P* = 0.35) (Figure 1B). The majority of patients diagnosed were older than 30 years (85%; Figure 1C). Gender distribution was roughly equal within the patient population (49% female, 51% male). Patient home residence was captured for 592 of 620 positive tests, with 82% (*n* = 485), indicating a home address in Maryland, 14% (*n* = 80) in Washington, DC, and 1% (*n* = ≤5) in Virginia. The first 3 digits of patient home zip codes were used to understand the geographic distribution of patients from the Baltimore–Washington metropolitan area (Supplemental Figure 1). The remaining 23 patients (4%) listed primary residences in 11 different states. A complete breakdown of patient demographics and clinical metadata are available in Supplemental Table 2.

### Sequenced samples and characteristics of the virus.

We performed whole genome sequencing on 143 samples from unique patients using residual RNA following the diagnostic PCR (Table 1). Samples were sequenced in 2 phases, with the first phase enriched for patients admitted to the ICU (55 samples collected March 11–21, containing 14 patients admitted to the ICU), and the second capturing as many samples as possible for sequencing, irrespective of disease severity (of 88 samples collected March 13–31, 10 were from patients admitted to the ICU).

**Figure 2:**
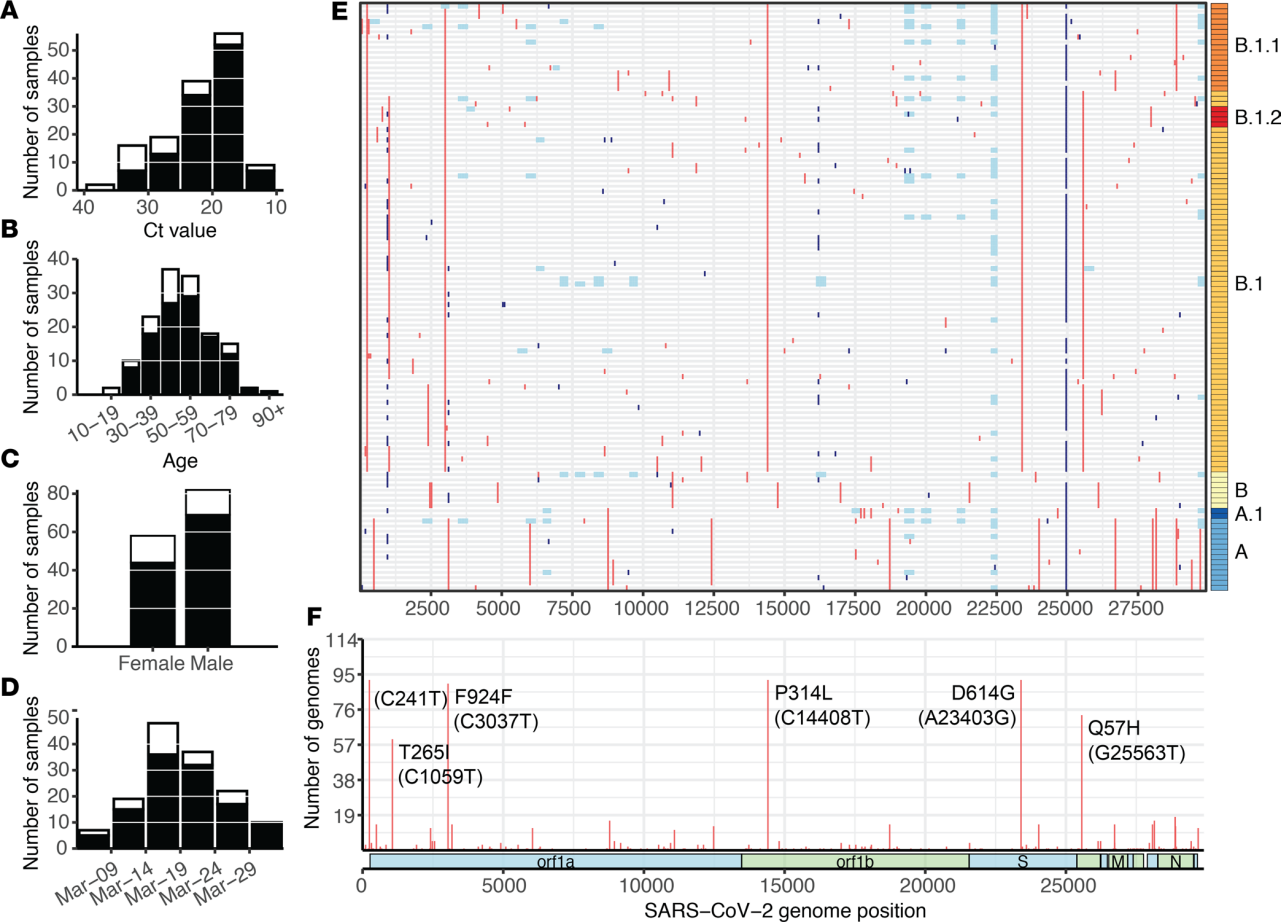
SARS-CoV-2 samples selected for whole genome sequencing. (**A**–**D**) Distribution of C_T_ value (**A)**, age (**B**), sex (**C**), and collection date (**D**) for specimens selected for whole genome sequencing (white bars), and specimens that produced complete genomes (black bars). Only specimens with known values are included in each plot. (**E**) Mutations across the SARS-CoV-2 genome in all 114 complete genomes (rows), binned into 60-nucleotide windows. Red, single nucleotide variant; light blue, base masked as N due to amplicon dropout; and dark blue, ambiguous base (N) due to variant-calling issues in homopolymer regions. Rows are clustered by Hamming distance between sequences and colored by Pango lineage (see Figure 3). (**F**) Count of complete genomes (out of 114) with a variant at each site. Key lineage-defining mutations are labeled. C_T_, threshold cycle.

We performed multiplexed pooled amplicon sequencing as described by the ARTIC network (23) on Oxford Nanopore instruments (GridION, MinION). From the 143 sequenced samples, we generated 114 complete genomes (76%), where complete genomes are defined as having at least 27,000 (out of 29,903) unambiguous nucleotides (see Methods). For validation, a subset of 31 samples were also sequenced on an Illumina MiSeq using the same amplicons (Supplemental Table 3). Sequenced samples ranged in C_T_ from 14 to 38. Of the samples with C_T_ values less than 30, 86% produced complete genomes, compared with only 39% of complete genomes from samples with higher C_T_ values (Figure 2A). This is consistent with other SARS-CoV-2 sequencing studies using the Oxford Nanopore platform (3, 24). We found no bias in our ability to generate complete genomes across key metadata categories, such as patient age, patient sex, and sample collection date (Figure 2, B–D).

The 114 complete genomes (from 114 distinct individuals) correspond to approximately 18% of JHHS-confirmed cases in March. Incomplete genomes were primarily due to amplicon dropout (26 of 34 failed samples), leading to stretches of ambiguous base calls across the genome that are also observed — to a lesser extent — in our complete sequences (Figure 2E). Despite these ambiguities, SARS-CoV-2 sequences can be grouped into clusters (or phylogenetic clades) based on a small number of variant sites in high-quality regions.

The 114 sequences were on average 98.6% complete, and we identified a total of 153 unique, unambiguous single nucleotide variants across all sequences (54 synonymous variants, 91 nonsynonymous variants, 8 noncoding variants) compared with the Wuhan-Hu-1 SARS-CoV-2 reference genome (accession no. MN908947.3), with a range of 2–14 variants per genome (Supplemental Table 4). In some samples, we observed a previously identified a cluster of 3 nucleotide mutations starting at position 28,881 that always occur together, resulting in 2 amino acid changes. Within the 114 complete genomes, 20 had 1 or 2 mixed sites (25%–75% alternate allele frequency), which we replaced with IUPAC ambiguity codes (25) because we were interested in consensus genomes for this study. Of 21 of these non-N ambiguities, 12 were at putative C-to-T mutations. We identified 5 clusters of sequences based on their polymorphisms, which correspond to phylogenetic clades (Figure 2E). Using the Pango nomenclature system developed by Rambaut et al. (14), we determined that the majority of our sequences (80%) belong to the B.1 lineage (Figure 2F and Supplemental Table 3). Within the established lineages (as of November 13, 2020), we identified 5 small groups of sequences from our data set (3–7 sequences per group) that share 2 or more additional single nucleotide variants (Supplemental Figure 2 and Supplemental Table 3).

### Variant validation.

We developed a rigorous bioinformatics pipeline to validate variant calls used to identify lineages and generally improve the quality of consensus genomes used for downstream analyses. The Oxford Nanopore platform has been widely used to generate SARS-CoV-2 data worldwide (24, 26, 27), but known issues identifying bases in low-complexity regions (28) may confound these data. To detect and correct possible errors, we first compared the results from multiple variant callers (Medaka, Nanopolish and a naive Samtools base caller; refs. 29, 30) and found Nanopolish to be most reliable for SARS-CoV-2 data, though our pipeline automatically calls variants with multiple callers and reports any discrepancies between them (29).

We also validated variants by employing negative controls (NTCs) to eliminate data with any evidence of contamination (see Methods) and by resequencing 31 of our 114 samples on the Illumina platform. Of the 280 consensus variants (including Ns) identified in these 31 samples using the Oxford Nanopore platform, all but 1 were validated by Illumina data. Looking more closely at allele frequencies, 251 sites had variant allele frequency greater than 75% in both technologies, whereas 28 sites had greater than 75% allele frequency in 1 technology but not the other. In every case, this allele frequency discrepancy occurred because of base calling issues in Oxford Nanopore reads in homopolymer regions. Over half of all homopolymer issues occurred at position 3037, which becomes a 5-nucleotide T-homopolymer and has previously (31) been identified as a likely problematic site (Supplemental Figure 3 and Supplemental Figure 4A). The single mismatched variant is in a low-sequence complexity region (position 27,673) and was at mixed frequency in Oxford Nanopore data (45%) and 100% frequency in Illumina. The low complexity of this region may have led to incorrect base calling in 1 of the 2 platforms (we also note that only 1 sample in our full data set has a variant at this site). However, the strong concordance between Oxford Nanopore and Illumina data at all other sites allows us to have high confidence in our sequencing data after accounting for homopolymer issues supported by other recent work (32).

In addition to base calling issues in homopolymeric regions, we also observed a strand-specific bias to Oxford Nanopore sequencing at some sites (Supplemental Figure 4B). This bias can occur when the sequence context is more difficult to base call in one direction, and is corrected in our pipeline by requiring that putative variant alleles are found on reads in both directions. It should be noted that both Nanopolish and Medaka consensus variant callers generate correct calls with sufficient coverage from both strands and this is only an issue if a significant imbalance in strand coverage arises.

Overall, we were able to show that known Oxford Nanopore issues (28, 32) do not affect SARS-CoV-2 lineage assignments, and we used our analysis to systematically correct common types of ambiguities (e.g., due to strand bias) in our bioinformatics pipeline, thus increasing the quality and completeness of our consensus genomes. We also note that the ambiguous base calls observed in most of our samples at positions 1001, 24981, and 24982 were due to similar issues in homopolymeric regions (Figure 2E, dark blue).

### Clinical correlates to viral genomics.

**Figure 3:**
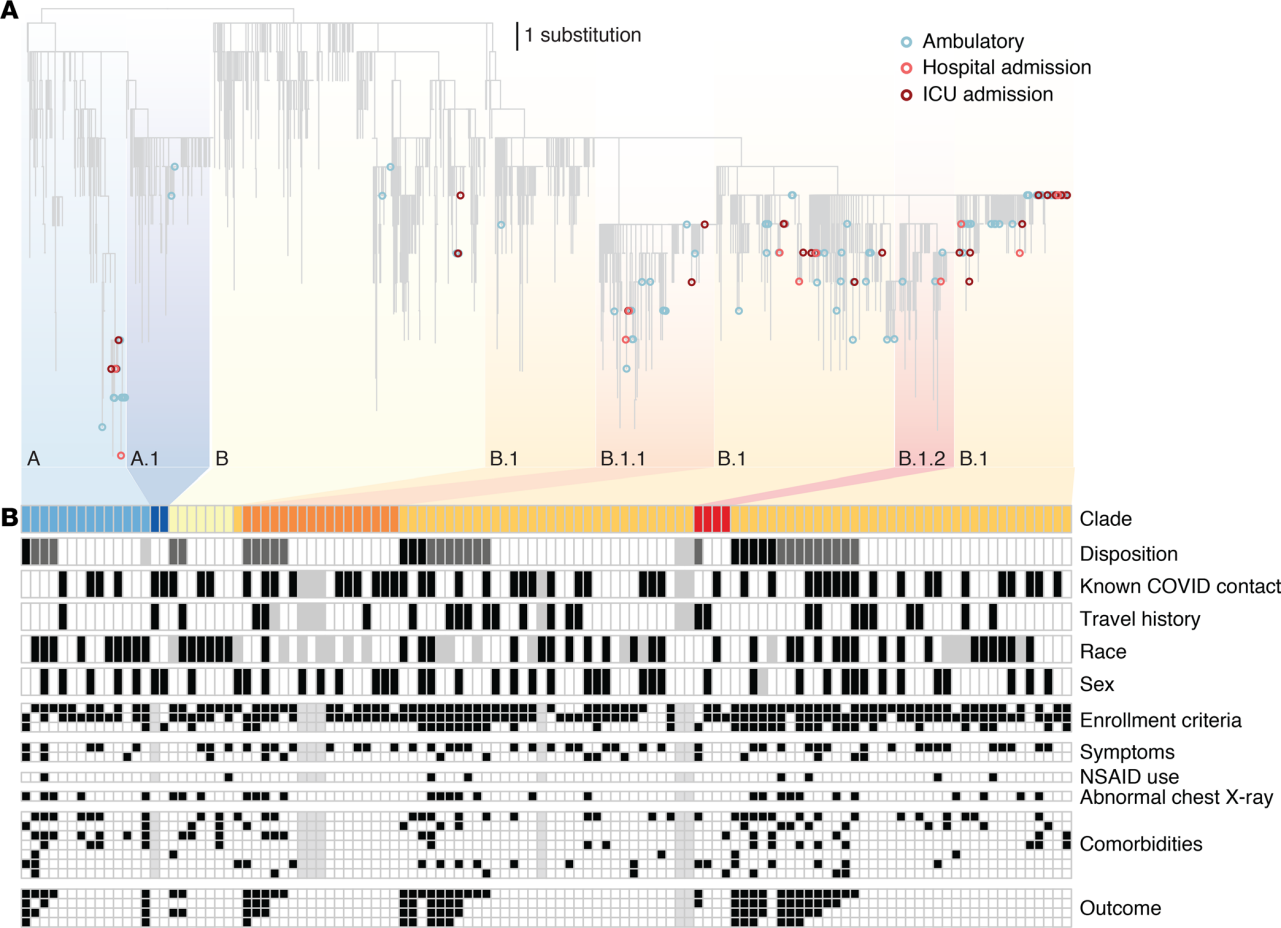
JHHS sequences and patient outcome. (**A**) Maximum likelihood tree of subsampled SARS-CoV-2 global data set and all 114 sequences generated in this study. Ambulatory (blue) includes all patients with no known admission to the hospital. Hospital admission (light red) includes admitted patients with no known admission to the ICU, including patients administered oxygen. (**B**) Clinical metadata and virus lineage. Each column represents 1 of the 114 patients with virus sequenced in this study, and columns are grouped by disposition within each lineage. Unless otherwise specified: black, yes; white, no; gray, unknown. Disposition: black, still in hospital or deceased as of May 15, 2020; dark gray, discharged; and white, never admitted. Race: black, Black; white, White; gray, other. “Other” includes < 10 each of American Indian/Alaska Native, Hispanic ethnicity (not otherwise specified as Black or White), other race not specified, or unknown. Sex: black, female; white, male. Enrollment criteria (top down): Fever, cough, and shortness of breath. Symptoms (top down): body ache, GI. Comorbidities (top down): cardiac disease, lung disease, diabetes, obese, alcohol, history of smoking (current and former smokers), and immunocompromised. Outcome (top down): hospital admission, supplementary oxygen, ICU admission, and ventilator administration. JHHS, Johns Hopkins Health System.

We performed in-depth chart reviews for all patients with samples selected for sequencing to evaluate potential relationships between the sequence of the virus and disease presentation. These chart reviews captured patient data including comorbidities, symptoms, and disease severity (Supplemental Table 2). We also looked for likely local transmission events, identified by the absence of reported travel in the 3 weeks prior to diagnosis, as well as likely travel-related importations from locations with known outbreaks in the same time period. In total, 32 (22%) had potential travel exposure from locations with early outbreaks, including the United Kingdom, California, Colorado, New York, and Idaho (travel history; Figure 3B), and 66 (46%) of patients reported having been potentially exposed in a high-risk scenario (Known COVID contact; Figure 3B). The 111 (78%) individuals that contracted the virus without reported travel history suggest that community transmission was occurring at this early stage of the pandemic.

Similar to larger studies (24), we observed a broad distribution of patient outcomes across the full diversity of SARS-CoV-2 mutations. We observed that severe cases, defined as admission to the ICU (including patients requiring ventilator support), had viral genomes spread throughout the phylogenetic tree and that belonged to each of the major SARS-CoV-2 lineages (A, A.1, B, B.1, B.1.1, B.1.2) observed globally (Figure 3A). Similarly, patient phenotypes including sex, race, recent travel, symptoms, and comorbidities were represented across these lineages, suggesting that susceptibility was independent of virus lineage (Figure 3B).

The widely examined mutation in the viral spike protein (D614G) has been proposed to have an effect on virus transmission (33–35). This mutation is one of the differentiators of the B.1 lineage (the A lineage and B lineages outside of B.1 and its sublineages do not have the mutation). In our data set, a similar proportion of patients infected with virus with and without the mutation had severe disease, again defined as admission to the ICU (21.7% vs. 20.2%; *P* = 0.87). However, our sample size of 114 is much smaller than the 301 per lineage (B.1 vs. other) that would be needed to detect a 10% difference in ICU admission rates. Thus, we are underpowered to show significant correlations between viral genome mutations and disease severity. That said, the diversity of clinical symptoms and patient outcomes observed in lineages spanning the global phylogenetic tree suggests that viral mutations are not the main driver of clinical presentation, as has been observed in larger studies with more power to detect correlations (36, 37).

### Evaluation of regional and global SARS-CoV-2 genetic diversity.

**Figure 4:**
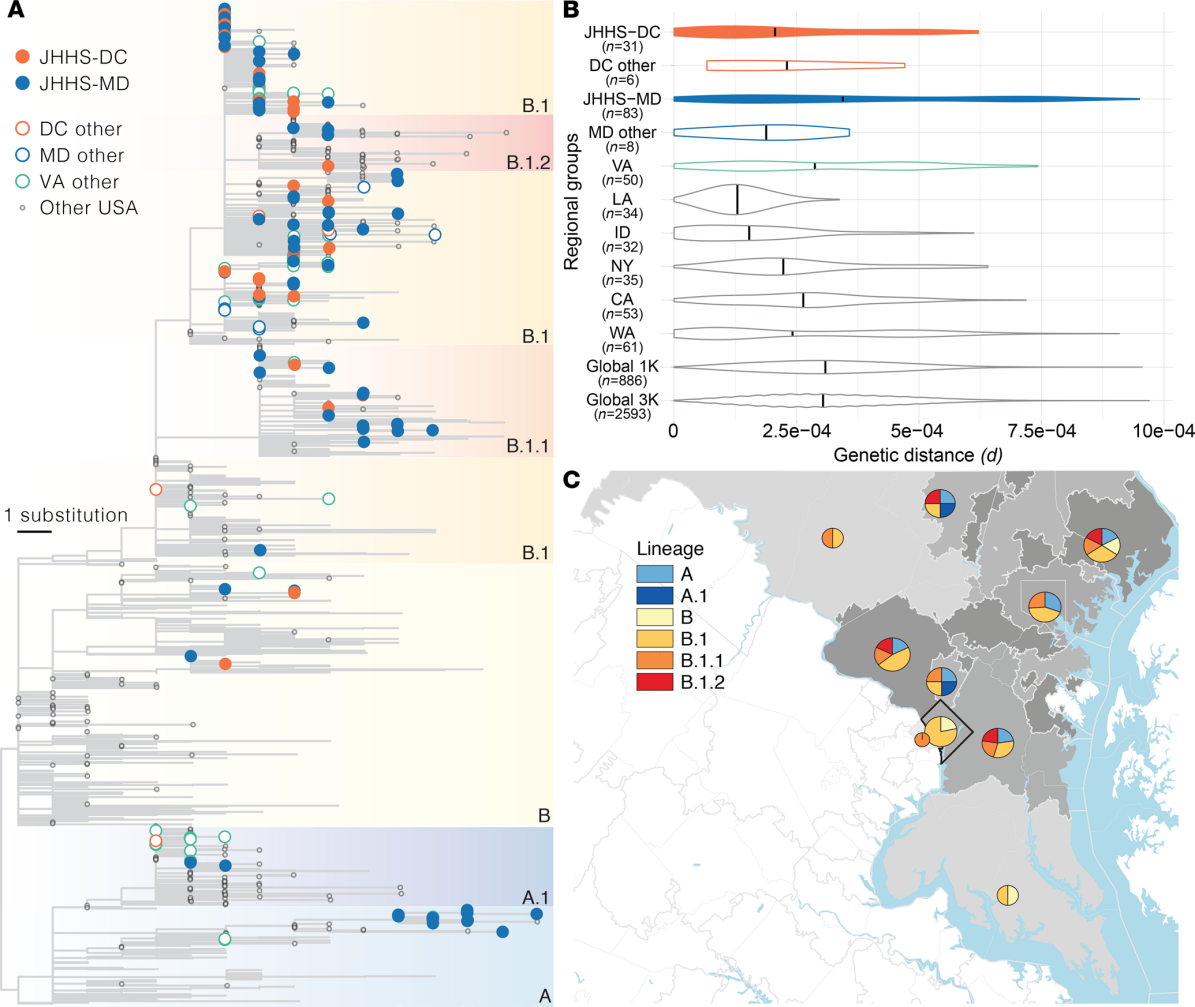
Geographical context of sequences from the Baltimore–Washington metropolitan area. (**A**) Maximum likelihood tree. Filled tips belong to sequences generated in this study. Major phylogenetic lineages (defined as lineages from the Pango nomenclature system (14) found in greater than 5% of samples in our subsampled global data set) are indicated by color blocks and labeled. (**B**) Evolutionary divergence in geographic groups. Violin plots represent the distribution of pairwise genetic distances between all sequences for samples collected in each listed geographic group. Colors are as in **A**, with filled violins containing sequences from this study. Black vertical lines depict the mean pairwise genetic distance between all samples in each regional group. (**C**) Map of the Baltimore–Washington metropolitan area. The number of sequences in this study with home locations in each area as defined by the first 3 digits of the patient zip code (ZIP3 area; Washington, DC outlined in black, all others gray) is indicated by shading of that region (darker, more sequences) and pie chart area. Pie charts show the proportion of sequences from each ZIP3 area belonging to each major lineage. Sequence counts between 1 and 5 are shown as 5 sequences. MD, Maryland; VA, Virginia; DC, District of Columbia; WA, Washington; CA, California; ID, Idaho; LA, Louisiana; NY, New York.

We compared our sequences from the Maryland and Washington, DC, region with others from around the world to better understand how the virus entered and spread within the region early in the outbreak. We performed phylogenetic analysis using JHHS-generated sequences and a globally representative reference data set containing all published sequences collected in Maryland, Washington, DC, and Virginia through the end of March 2020 (Figure 4A and Supplemental Table 6). We see that sequences from this region fall throughout the phylogenetic tree, and belong to both the major A and B lineages, as well as major global sublineages A.1, B.1, B.1.1, and B.1.2 (we defined major lineages as lineages or sublineages occurring in >5% of our global data set, see Methods). Bootstrap values throughout the tree are low (Supplemental Figure 6) due to minimal accumulated diversity early in the outbreak, but the structure of the tree suggests there were likely 5 or more separate introductions during the first few weeks. Within each observed lineage, we see groups of highly similar or even identical sequences (Figure 2E), suggesting that community transmission followed the initial introductions.

We also looked specifically at viral genetic diversity within the Baltimore–Washington metropolitan area compared with the total genetic diversity observed in other regions in the United States and around the world. We found that the distribution of and maximum average pairwise viral diversity observed between sequences in the JHHS Maryland data set were comparable with that of global sequences (JHHS Maryland maximum pairwise diversity = substitutions per site; global subsampling =; see also Supplemental Table 6 and Supplemental Table 7), concordant with our observation that regional sequences belong to all major globally circulating clades (Figure 4B and Supplemental Figure 5). This observation may reflect the national and international connectivity of the entire greater Washington, DC, area, as well as the travel patterns of individuals in this region, which includes 2 major metropolitan areas and 3 international airports.

Even within Maryland, the JHHS data set (JHHS-MD) had higher mean pairwise genetic distance than other data from the state (MD other). Other published sequences from Maryland were submitted by public health laboratories, and lower genetic distances may reflect sequencing of connected clusters of cases during outbreak investigation. The average pairwise genetic distance is lower in other parts of the region (Washington, DC, and Virginia) than in Maryland, though it is clear that there are sequences from multiple lineages present in both locations. It is unclear if the lower observed average distance in both JHHS and other sequences from Washington, DC, is a reflection of movement patterns of individuals within that nearby area or simply lower sample size (only 32 sequences from Washington, DC, are in our data set, compared with 82 total sequences from Maryland). Published sequences collected in Virginia suggest its genetic diversity falls between that of Maryland and Washington, DC; but Virginia is a large state, and without more detailed location information for these sequences, it is difficult to determine if the sequences truly represent the diversity circulating in the state as a whole (Figure 4B and Supplemental Figure 5).

Examining diversity from other US states that experienced outbreaks early in the epidemic, we see that the distribution of viral diversity in Maryland and Washington, DC, looked very different from diversity in states such as Louisiana and Idaho, which show very low mean genetic diversity during this period. This could be due to sampling of specific clusters of cases as described above (e.g., cases from a ski resort outbreak in Idaho; ref. 38), or could occur if early cases in these states were seeded by a single source. This stands in contrast to the outbreaks in Washington state and New York, for which sequence data clearly shows multiple introductions (5). As expected, the diversity of the viral population (the full distribution of pairwise genetic distances) in New York appears to be similar to that of the Baltimore–Washington metropolitan area, as the outbreaks in both regions were seeded multiple times and contain sequences predominantly belonging to the B.1 lineage (Figure 4, A and B, and Supplemental Figure 5).

Finally, we examined viral genetic diversity within and across our region, separating this area into subregions defined by the first 3 digits of their zip code (“ZIP3 location”). We found that 3 ZIP3 locations with the highest number of cases in Maryland and Washington, DC, each had sequences from multiple lineages, and that all but 1 ZIP3 location had sequences from more than 1 lineage (Figure 4C). We do not observe distinct segregation of viral lineages to particular locations within the region, which highlights both the rapid spread of the virus early in the pandemic and the interconnectedness of this region.

## Discussion

Our genomic data set from the Baltimore–Washington metropolitan area revealed diversity approaching that of the worldwide phylogeny, even in an early phase of the epidemic. Sequences from the region spanned the global phylogeny, suggesting multiple and diverse introductions from regional or international locations. We also observe minimal diversity within each of these specific lineages, suggesting transmission of the virus within local communities after an initial introduction. This pattern of diversity highlights the connectedness of the region to both the national and global epidemic, and the challenges that confront any control strategy.

The diversity we observe within the region is also visible on smaller geographic scales, with multiple viral lineages represented within each ZIP3 location in Maryland and Washington, DC. This suggests significant movement of viral lineages within the Baltimore–Washington metropolitan area before regional closures were implemented at the end of March, likely due to local transport and a large number of commuters between Maryland and Washington, DC. Further research on more recent COVID-19 cases will be needed to understand how national-, state-, and city-based regulations limiting travel and implementing physical distancing recommendations affected these patterns of spread as well as the impact of subsequent easing of these restrictions.

The diversity of sequences within this region, combined with detailed clinical metadata obtained through the JHHS, allowed us to explore the relationship between the SARS-CoV-2 virus and patient presentation and outcome. Specifically, we looked for viral genotypes that demonstrated a connection to disease severity, comorbidities, or patient demographics such as gender and race. We found no clear correlation, but were limited by sample size. It will be important to continue to analyze genomic data alongside clinical metadata as the number of available viral genomes increases to look for potentially subtle associations between the viral genome and patient characteristics (39).

The analyses described above rely on complete and accurate SARS-CoV-2 sequences. We used a tiled amplicon sequencing approach on the Oxford Nanopore platform to generate sequencing data and found that we were able to achieve complete genomes for a substantial portion of samples attempted. As in previous studies (24), we found that samples with higher virus titer (low C_T_ value) more reliably produced complete genomes, and these values can be used to triage samples for sequencing when resources are limited. We also observed some correlation between days from symptom onset and C_T_ value, suggesting that epidemiological surveillance may be most effective if patients are captured early in their course of infection.

We also performed validation on our sequences by using multiple variant callers to detect variants and sequencing a subset of samples on the Illumina platform. We found that amplicon-based sequencing with Oxford Nanopore generates correct consensus genome sequences (compared with Illumina sequences), but with some added ambiguities in specific problematic regions, especially homopolymers. We have developed a pipeline that corrects and flags these issues, and it is our hope that highlighting them in this paper contributes to the overall quality of SARS-CoV-2 sequences generated with this widely used platform that enables rapid sequencing in a variety of settings.

Moving forward, the pipelines established here will be critical to using genomic surveillance to inform the COVID-19 public health response. When confronting a new disease, the first genomes are the hardest to generate, as they require establishment of laboratory protocols and bioinformatic pipelines that can provide accurate and timely information. This has occurred in record time during the COVID-19 pandemic; the methods and results presented here will serve as the foundation of continued molecular surveillance of SARS-CoV-2 within the JHHS. Ongoing work will allow us to answer critical questions about not only the evolution of the virus but also the fundamental mechanisms by which control measures affected its epidemic spread. These efforts complement the information provided by the rapidly growing public databases of SARS-CoV-2 sequences by focusing the collection of genomic data in settings where we can access extensive current and past clinical data to investigate fundamental questions about this evolving virus’s changing relationship with human health.

## Methods

### Data availability.

Raw nanopore and Illumina data are deposited at SRA (BioProject PRJNA629390). Consensus sequences are deposited at GISAID and Genbank (MT509452-MT509493, and MT646048-MT646120) under BioProject PRJNA650037 (accession numbers available in Supplemental Table 3).

### Specimens and patient data.

Clinical specimens used for genetic characterization were remnant nasopharyngeal swabs available at the completion of standard of care testing at the Johns Hopkins Hospital clinical virology laboratory. In total, 143 samples were selected for analysis based on their distribution throughout March 2020 and representation of the range of disease severity observed during this period. During this period, automated patient metadata extraction was limited to the date a sample was confirmed positive; all other data required patient chart reviews. Samples were sequenced in 2 phases, with the first phase enriched for patients admitted to the ICU (14 of 55 samples collected March 11–21), and the second a convenience sample that captured as many samples as possible for sequencing, irrespective of disease severity or ICU admission (10 of 88 samples collected March 13–31).

### Clinical data analysis.

Data including patient demographics, symptoms, comorbidities, COVID-19 exposure, recent travel history, and results of chest imaging at presentation were abstracted from the electronic medical record (EMR). COVID-19 treatment (medication, supplemental oxygen, and invasive mechanical ventilation) and outcomes (home observation without inpatient admission, discharge after admission, ongoing admission, and death) were also abstracted from the EMR. Race as self-reported by the patient and documented in the EMR was collected in prespecified categories. Patients who reported (a) contact with an individual known to be COVID-19–infected or (b) high-risk exposure (e.g., healthcare worker) were classified as COVID-19–exposed. Comorbidities were assessed based on diagnoses in the EMR (i.e., diabetes, obesity, or alcohol use disorder) and further categorized for lung disease (e.g., asthma, COPD), cardiac disease (e.g., valvular heart disease, arrhythmias, hypertension), and immunocompromised (e.g., HIV positive, hematologic malignancy, solid organ transplant).

### Nucleic acid extraction.

Automated nucleic acid extraction was performed using either the NucliSENS easyMag or eMAG instruments (bioMérieux) using software version 2.1.0.1. easyMag or eMAG lysis buffer (2 mL) was added to 500 μL of aliquoted viral transport media in a biosafety cabinet in either a BSL-3 or BSL-2 facility using BSL-3 biosafety measures. Specimens were incubated for 10 minutes in the lysis buffer prior to automated nucleic acid extraction following the off-board lysis bioMérieux protocol, with an RNA elution volume of 50 μL.

### Diagnostic reverse transcription PCR (RT-PCR).

The Altona Diagnostics RealStar SARS-CoV-2 RT-PCR Kit 1.0 was the primary assay used for molecular diagnosis. A subset of SARS-CoV-2 positives were identified using the Cepheid Xpert Xpress SARS-CoV-2 GeneXpert platform per manufacturer instructions. All samples were processed within 24 hours of collection.

The RealStar SARS-CoV-2 RT-PCR Kit 1.0 total reaction volume was 30 μL (10 μL extracted sample and 20 μL Master Mix). The kit contains 2 premade master mixes, A and B, which contain PCR buffer, magnesium salt, primers and probes, reverse transcriptase, and DNA polymerase. The detectors used are Cy5 (SARS-CoV-2; S gene), FAM (B-βCoV; E gene), and JOE (Internal Control). C_T_ values for the S gene target (Cy5) are reported in Supplemental Table 2. Taqman RT-PCR was performed using the Prism 7500 Sequence Detection System (Applied Biosystems) at the following cycling conditions: 1 cycle at 55.0°C for 20 minutes, 1 cycle at 95.0°C for 2 minutes and 45 cycles at 95.0°C for 15 seconds, 55.0°C for 45 seconds and then 72.0°C for 15 seconds. Validation of this assay was performed as described in ref. 22.

Testing on the Cepheid Xpert Xpress SARS-CoV-2 GeneXpert platform was performed in accordance with manufacturer’s instructions (40).

### Genome sequencing with ARTIC tiled amplicons.

Whole genome amplification of the SARS-CoV-2 genome was performed using a modified ARTIC network protocol with the V3 primer set (23). Briefly, cDNA was generated from previously extracted RNA remaining after the initial diagnostic RT-PCR assay. No sample dilution was performed to normalize samples by C_T_ value ranges, as these data were often incomplete at the time of sample processing. A 2-step reverse transcriptase PCR was performed using random hexamer cDNA synthesis using SuperScript IV (Thermo Fisher, 18091), followed by multiplexed PCR in 2 nonoverlapping pools using Q5 DNA polymerase (New England Biolabs, M0491). For Oxford Nanopore sequencing, amplicon pools were indexed using the Native Barcoding reagent set (Oxford Nanopore, EXP-NBD104). Indexed sample sets of 11 were then pooled, and 20 ng of the resulting library was used for sequencing on Oxford Nanopore GridION instruments using R9.4.1 flow cells and high-accuracy basecalling (Guppy v3.5.2).

For Illumina sequencing, the New England Biolabs NEBNext Ultra II DNA Library Prep Kit for Illumina reagent set was used for library generation from the same starting amplicons as used for Oxford Nanopore library preparation. Sequencing adapters were diluted 10-fold for our input range of 5–100 ng of DNA. Adapter-ligated DNA was cleaned up without size selection and underwent 8 cycles of PCR at the amplification step. Samples were sequenced on a MiSeq using a 600bp v3 cartridge.

### Genome assembly and variant validation.

Reference-based genome assembly was performed using the ARTIC network bioinformatics pipeline v1.0.0 for COVID-19 (https://artic.network/ncov-2019) with modifications. Briefly, base called reads were demultiplexed with Guppy v3.5.2. Reads were mapped to the SARS-CoV-2 reference (GenBank accession MN908947.3) with minimap2 v2.17 (41) and coverage was normalized across the genome using a custom normalization pipeline (https://github.com/mkirsche/CoverageNormalization; https://zenodo.org/record/4450293#.YCaj9xNKjSw) (42) with coverage_threshold 150 and parameters --even_strand and --qual_sort. Primer binding regions were masked and variant calling was performed with Nanopolish v0.13.2 with a minimum candidate allele frequency of 0.15 (43). Consensus genomes were generated with bcftools v1.9 (44) by mapping called variants to the reference genome, and all sites with less than 20x coverage were masked as “N.”

A custom pipeline was used to validate called variants (https://github.com/timplab/jhu-covid-pipeline; https://zenodo.org/record/4453269#.YCak7hNKjSw) (45). This pipeline made use of the NTC on each sequencing run. Amplicon regions with 1 or more positions with read depth less than 2 times the 95% quantile of average amplicon depth in the NTC (minimum threshold = 20) were masked as “N,” and any variants also present in the NTC were masked unless the coverage at that variant position was more than 5 times the NTC coverage at that position. Additionally, any sequencing runs with high coverage in the NTC (>50x depth threshold) were ignored and all samples rerun.

Variants in samples or regions without evidence of contamination were validated by 2 other variant callers: Medaka v0.11.5 (implemented by re-running the ARTIC bioinformatics pipeline) and a naive Samtools (44) variant caller, both with a minimum candidate allele frequency of 0.15. All positions with variant caller disagreements or high minor allele frequencies (mixed variants; variant allele frequency 25%–75%) were manually inspected in Integrated Genome Viewer (46). Mixed variants found only on 1 sequencing strand were ignored (called as reference base), and mixed variants due to deletions in clear homopolymer regions were called as the alternate allele in the consensus genome. For the purposes of creating a consensus genome, candidate variants at less than 25% allele frequency were called as the reference base, and variants greater than 75% frequency were called as the alternate allele.

When available, Illumina data were used to confirm or invalidate variants with disagreements. The same normalization process was applied to Illumina reads, and variant calling was performed with FreeBayes v0.9.21 (47), iVar v1.0 (48), and Samtools. Mixed variants that could not be confirmed with Illumina data or (in)validated due to strand bias or homopolymer deletions were replaced with ambiguity codes. Final variants were annotated with SnpEff (49).

Genomes were considered complete if they had at least 27,000 non-N nucleotide calls (specific IUPAC ambiguity codes such as Y or R contributed to reaching the 27,000 threshold). We also required that the sequence had fewer than 5 mixed variants, as the cause of highly mixed samples (perhaps due to contamination, coinfection, or within-host variation) requires further research.

Genomic analyses were performed on the SciScerver science platform (50).

### Correlating viral diversity to clinical characteristics.

Complete patient records were available for 112 of the 114 virus genome sequences evaluated in this study. χ^2^ tests were performed to evaluate the correlation between severe disease, defined as ICU admission, and the presence or absence of the D614G mutation.

Power calculations were performed using the overall ICU admission rate (21%) to determine the total number of patients in relevant clades to detect a 10% difference in disease severity at 80% power. A total of 301 patients per mutation (602 total) were determined to be necessary to differentiate the impact of the D614G mutation on disease severity. With the genetic distribution of the 112 samples analyzed in this study, the power to determine a 10% difference in association between virus genotype and disease severity was estimated at 12%.

### Selecting a genomic background data set.

For phylogenetic analyses, full-length viral genome sequences with collection dates before April 1, 2020, were downloaded from Genbank (51) and GISAID (18) on June 3, 2020. Multiple sequence alignment was performed using MAFFT v7.458 (52) using parameters --reorder --anysymbol --nomemsave --adjustdirection. Sequences with fewer than 75% unambiguous bases were excluded, as were duplicate sequences defined as having identical nucleotide composition and collected on the same date and in the same country. The resulting data set was trimmed at the 5′ and 3′ ends resulting in a multisequence alignment with 29805 nucleotides. This data set was then subjected to multiple iterations of phylogeny reconstruction using IQ-TREE multicore software version v1.6.12 (53) with parameters -m GTR+G -nt 50, and exclusion of outlier sequences whose genetic divergence and sampling date were incongruent using TempEst (54), resulting in a data set with 19,565 sequences.

For computational efficiency, we downsampled this data set homogeneously through time and space, by randomly selecting 7 and 34 sequences per month, to obtain global data sets with 1168 (hereafter referred to as Global 1K) and 3113 (hereafter referred to as Global 3K) sequences from around the world, respectively. We preferentially selected longer sequences with the fewest number of gaps in the 5′ and 3′ ends and those that had complete dates and the fewest number of ambiguous bases. We used the high-performance computational capabilities of the Biowulf Linux cluster at the NIH (http://biowulf.nih.gov) to perform these downsampling analyses. After downsampling, we removed any sequences with fewer than 27,000 unambiguous bases and any remaining sequences deemed GenBank/GISAID duplicates.

To study regional epidemics within the United States, sequences from Washington (WA), California (CA), Idaho (ID), Louisiana (LA), and New York (NY) were excluded from the Global 1K and 3K data sets. Data from the greater Washington, DC, region (Maryland, Washington, DC, and Virginia) were also removed from these global data sets, resulting in final global data sets of 886 (Global 1K) and 2593 (Global 3K) sequences (Supplemental Table 5 and Supplemental Table 6).

### Comparison of evolutionary divergence.

We estimated the evolutionary divergence of several sequence data sets from each of the locations selected for regional analysis. Each regional data set consisted of sequences removed from the Global 3K data. For Maryland and Washington, DC, we supplemented these to include all 114 JHHS sequences. We then subsetted these sequences based on whether they were generated as part of this study (JHHS-MD and JHHS-DC) or from other laboratories (Maryland other, Washington, DC other). For Virginia, we supplemented the removed sequences to include all Virginia sequences in the pre-downsampled global data set. The final regional data sets were as follows: JHHS-DC (*n* = 31); DC other (*n* = 6); DC = JHHS-DC + DC other (*n* = 37); JHHS-MD (*n* = 83); MD other (*n* = 8); MD = JHHS-MD + MD other (*n* = 91); VA (*n* = 50); LA (*n* = 34); ID (*n* = 32); NY (*n* = 35); CA (*n* = 53); WA (*n* = 61) (Supplemental Table 6).

To estimate evolutionary divergence, we calculated the pairwise divergence (in base substitutions per site) between all pairs of sequences within and between each geographical group. We conducted the analyses through the Molecular Evolutionary Genetics Analysis software version 10 (MEGA X) (55, 56) and applied the maximum composite likelihood mode (57). The rate variation among sites was modeled with a γ distribution (shape parameter = 4), and the differences in the composition bias among sequences were considered in evolutionary comparisons (58). We included 1st+2nd+3rd+noncoding codon positions, and all with less than 50% site coverage, due to alignment gaps, missing data, and ambiguous bases, were eliminated (partial deletion option).

We included the Global 1K and 3K data sets in this analysis to determine the appropriate global reference data set (out of more than 60,000 global SARS-CoV-2 sequences published at the time of analysis) for our phylogenetic analyses. Despite the significant difference in number of sequences, the Global 1K and 3K data sets we tested have comparable mean pairwise genetic distances, indicating that the smaller 1K data set is representative of global diversity and is an appropriate selection of sequences to use as the background for our phylogenetic inference (Supplemental Figure 5, Supplemental Table 5, and Supplemental Table 6).

### Phylogenetic analysis.

We used a customized Nextstrain Snakemake pipeline (12) using augur v9.0.0 on the final data set, which included the 886 Global 1K data set plus all removed regional groups (including all 114 JHHS sequences and all published Virginia sequences), resulting in 1279 sequences used in our phylogenetic analysis. We computed the phylogeny with IQ-TREE v1.6.12 (53, 59) with parameters -me 0.05 -nt 4 -m GTR -n 4. Trees were rooted on the Wuhan-Hu-1 reference genome in FigTree (60) and visualized using ggtree (61) in R (62). Finally, clades were assigned to sequences using Nextstrain (15) and the Pango nomenclature system (14) (see Supplemental Table 3). The lineages assigned by the Pango nomenclature system were used to discuss viral diversity throughout this manuscript, and we defined major lineages (A, A.1, B, B.1, B.1.1, B.1.2) as lineages or sublineages appearing in at least 5% of the sequences in our global subsample. Sublineages were collapsed into these 6 lineages by assigning the closest ancestral lineage that belongs to this group (e.g., B.1.37 becomes B.1, whereas B.3 becomes B).

### Statistics.

Continuous variables were categorized and descriptive statistics used to characterize the study population with respect to demographics, travel history, comorbidities, symptoms and disease severity. Proportions were compared using a χ^2^ test. A *P* value of less than 0.05 was considered to be significant. Power calculations were conducted for a 2-sample proportions test, based on assumptions of a 5% false-positive (i.e., α) rate and power of 0.8. Analyses were performed using Stata version 14 (StataCorp).

### Study approval.

All research was conducted under Johns Hopkins IRB protocol IRB00221396, which allowed the analysis and presentation of results under a waiver of consent.

## Author contributions

PMT, JL, LS, SCR, WT, and HHM designed the research study. JL, MCS, JDE, SCR, WT, and HHM coordinated the collaboration. PMT, NS, YF, VG, and HHM acquired the data. PMT, TM, OFN, CPM, MH, MS, SR, and LS collected and incorporated clinical data. SW, TM, SCR, MK, NST, AE, CH, CPM, YF, SR, and MCS analyzed sequencing data. PMT, SW, NST, LS, JDE, SCR, and WT wrote the manuscript. All authors reviewed and approved the manuscript. PMT and SW contributed equally, with PMT leading and designing the research study, and SW led the analysis efforts.

## Supplementary Material

Supplemental data

Supplemental Table 1

Supplemental Table 2

Supplemental Table 3

Supplemental Table 4

Supplemental Table 5

Supplemental Table 6

Supplemental Table 7

## Figures and Tables

**Table 1:**
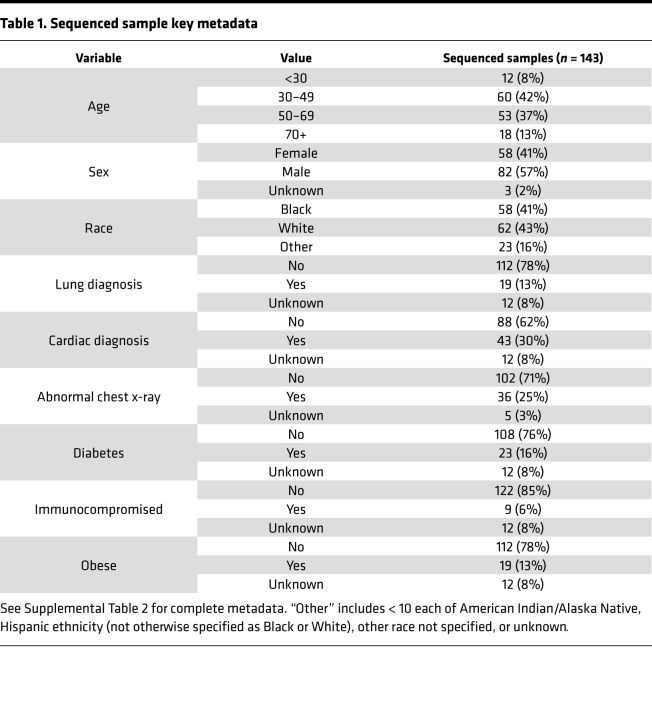
Sequenced sample key metadata

## Version 1 03/22/2021

Electronic publication

## Version 2 03/27/2021

Supplemental tables were added.
