# Supplementary material for: Genomic diversity of SARS-CoV-2 during early introduction into the Baltimore–Washington metropolitan area
Source: JCI Insight. 2021 Mar 22;6(6):e144350. doi: 10.1172/jci.insight.144350 (PMC8026189; doi:10.1172/jci.insight.144350)
Supplement: Supplemental data [file jciinsight-6-144350-s213.pdf]

Supplemental Figures

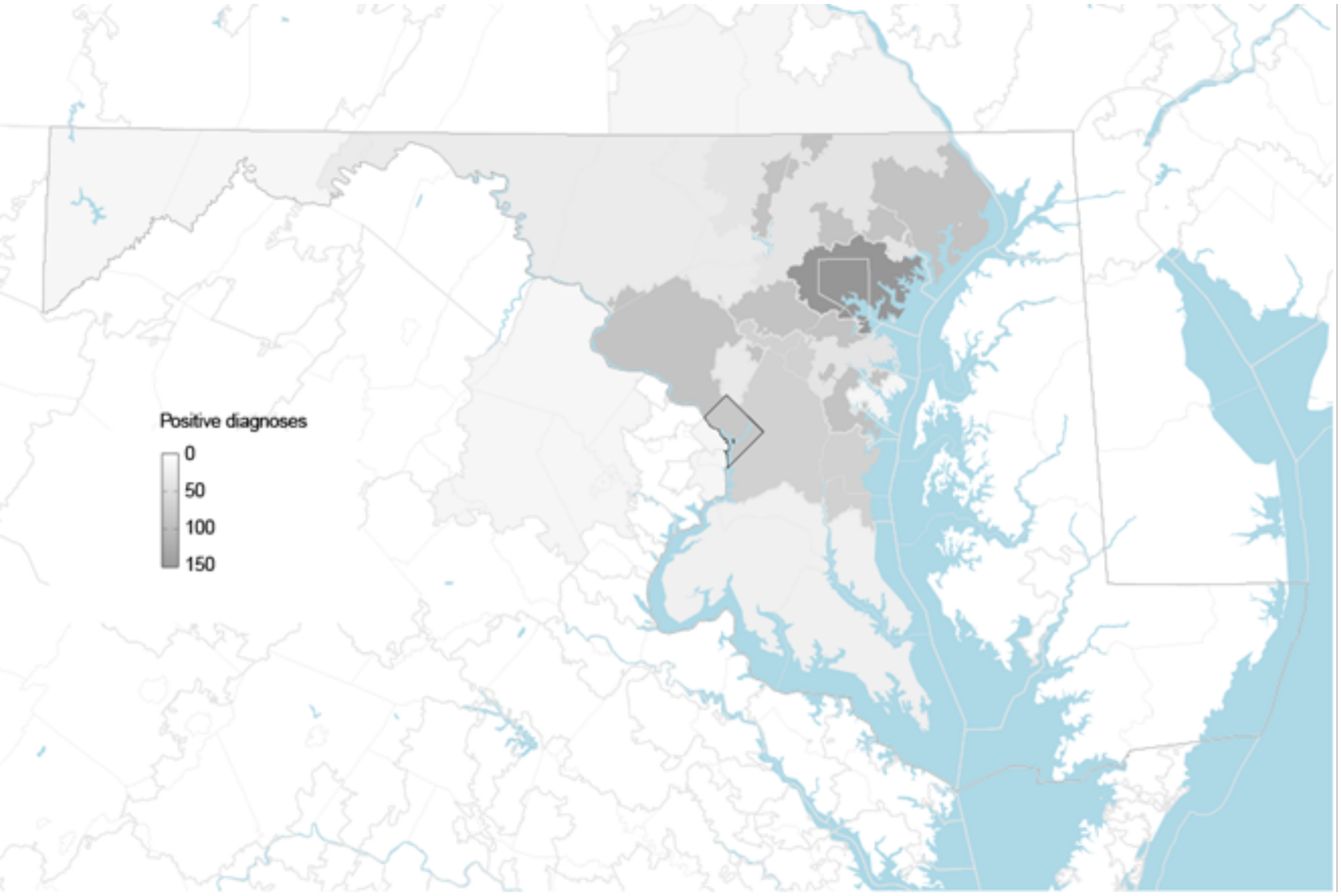

Supplemental Figure 1. Positive diagnoses by first three digits of zip code. Outlined regions correspond to areas sharing the first three digits of their zip code (Washington, DC outlined in black, all others grey). Each region is shaded by the number of patients with positive COVID-19 diagnoses in March 2020 (from the Johns Hopkins Health System) reporting home residence in that region. Regions with 1–5 positive diagnoses are shown as 5.

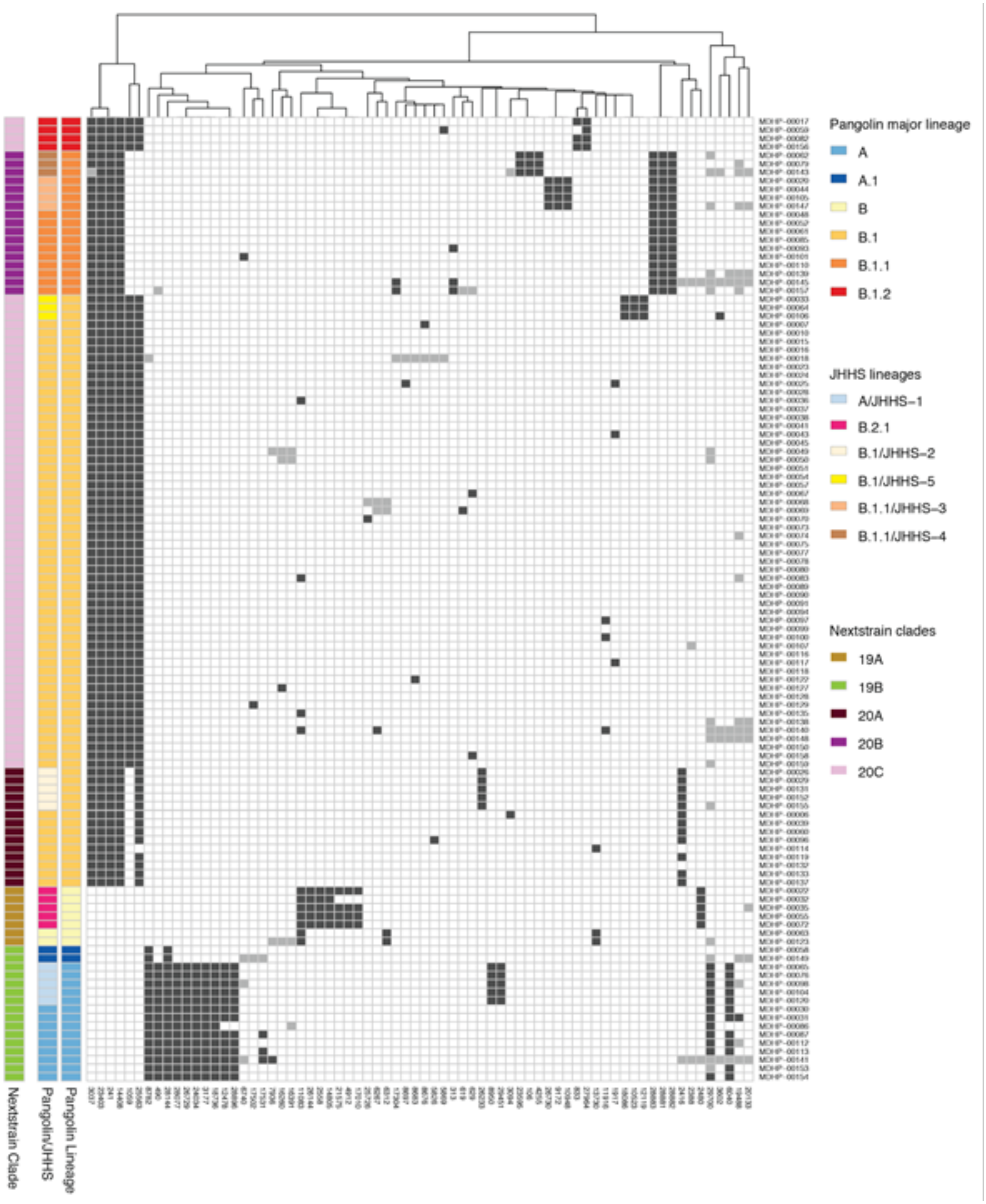

Supplemental Figure 2. Single nucleotide polymorphisms present in three or more JHHS sequences. Rows = samples sequenced as part of this study (114), sorted by Pangolin lineage. Columns = positions with consensus variants in three or more sequences. Black cells = variant present; grey cells = ambiguity present. Columns are clustered using complete linkage hierarchical clustering. JHHS lineages are sub-groups of pangolin lineages in which two or more variants are shared between three or more JHHS sequences. In one case (B.2.1), the JHHS sub-lineage is captured by a non-major pangolin lineage designation.

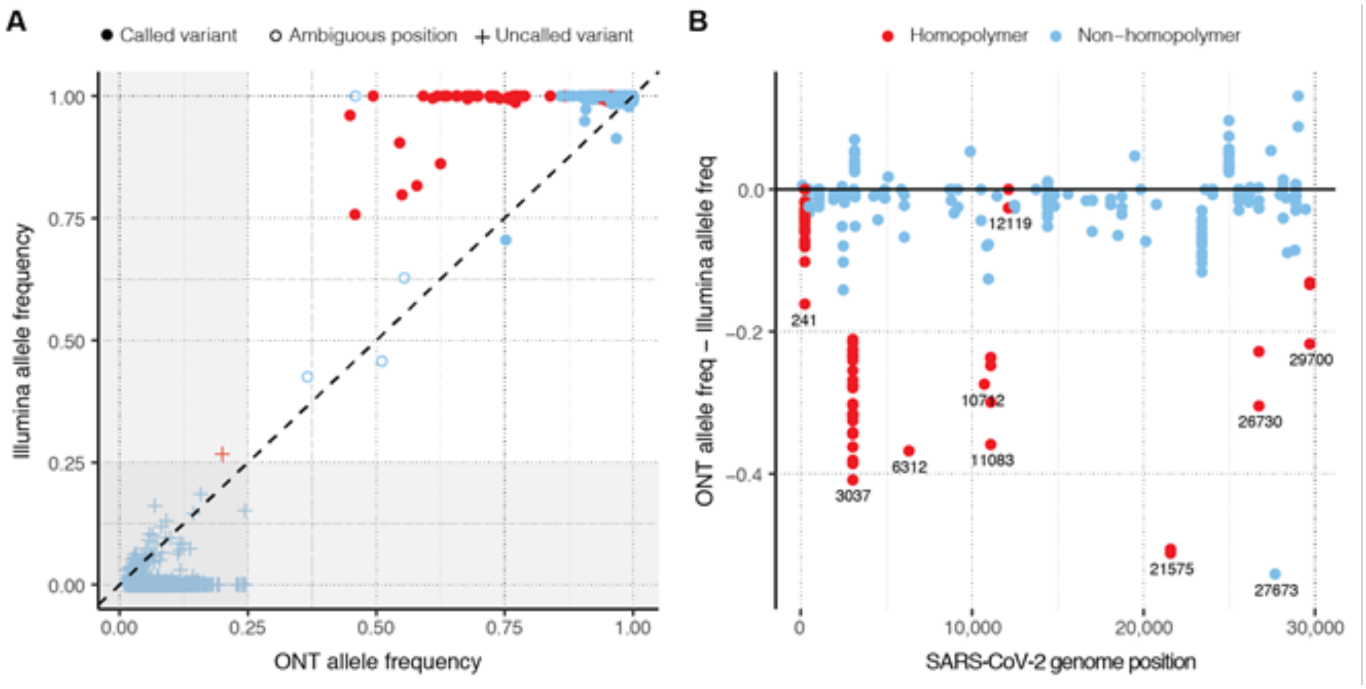

Supplemental Figure 3. Illumina and Oxford Nanopore allele frequency comparison. (**A**) Variant frequencies from tiled amplicons sequenced on the Oxford Nanopore (ONT) and Illumina platforms. All variants at >0.02 allele frequency are shown. Uncalled variants are those not present in the consensus sequences from these samples. Grey shaded regions represent frequency threshold (25%) used to automatically reject candidate variants. Almost all discrepancies in frequency occur within homopolymer regions (red symbols). (**B**) Difference between allele frequencies across the two sequencing platforms. Position 3,037 (within a T-homopolymer) accounts for most large discrepancies in frequency.

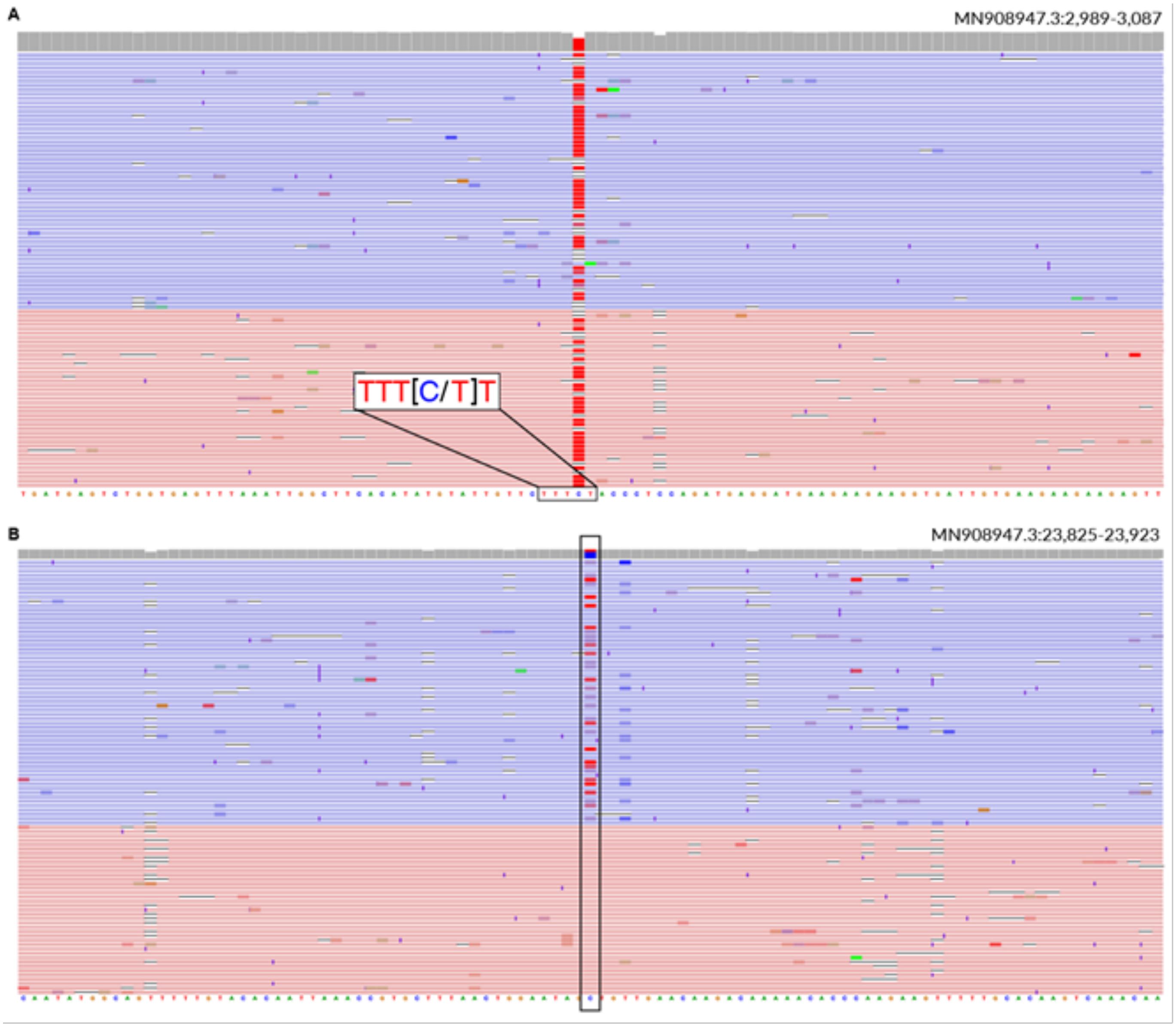

Supplemental Figure 4. Common Oxford Nanopore sequencing issues. (**A**) Example of a T-homopolymer region in the SARS-CoV-2 genome (sample MDHP-00028). A large number of reads have deletions, resulting in a mixed alternate allele frequency. (**B**) Example of strand bias in Oxford Nanopore sequencing reads (sample MDHP-00028). A mutation only occurs in minus strand reads.

Genetic distance (d) ($x10^{-4}$)

	JHHS-DC (n=31)	DC other (n=6)	DC (n=37)	JHHS-MD (n=83)	MD other (n=8)	MD (n=91)	VA (n=50)	LA (n=34)	ID (n=32)	NY (n=35)	CA (n=53)	WA (n=61)	Global 1K (n=886)	Global 3K (n=2593)
**JHHS-DC**	**2.25**													
**DC other**	2.10	**2.00**												
**DC**	2.17	2.03	**2.20**											
**JHHS-MD**	3.13	2.88	3.09	**3.73**										
**MD other**	2.23	2.07	2.21	3.16	**1.83**									
**MD**	3.05	2.81	3.01	3.64	3.03	**3.62**								
**VA**	2.77	2.47	2.72	3.47	2.73	3.41	**2.95**							
**LA**	2.19	2.09	2.18	3.17	2.19	3.08	2.89	**1.32**						
**ID**	2.32	2.15	2.29	3.26	2.29	3.17	2.93	1.68	**1.52**					
**NY**	2.47	2.26	2.43	3.34	2.42	3.26	3.02	2.39	2.49	**2.51**				
**CA**	3.06	2.61	2.99	3.62	3.21	3.59	3.13	3.38	3.36	3.31	**2.64**			
**WA**	4.20	3.39	4.07	4.53	4.43	4.52	3.84	4.62	4.55	4.41	3.11	**2.57**		
**Global 1K**	3.09	2.79	3.04	3.71	3.24	3.67	3.31	3.43	3.41	3.37	2.98	3.74	**3.16**	
**Global 3K**	3.00	2.73	2.96	3.65	3.13	3.60	3.27	3.31	3.30	3.28	3.04	3.86	3.17	**3.16**

Supplemental Figure 5. Evolutionary divergence in geographic regions. Pairwise matrix containing average pairwise genetic distances within (bolded in the diagonal) and between sequences from various geographic regions including: the District of Columbia (JHHS-DC + DC other), Maryland (JHHS-MD + MD other), Virginia (VA), Louisiana (LA), Idaho (ID), New York (NY), California (CA), Washington (WA), and two representative global subsamples (Global 1K and 3K). Green-to-red color scale highlights the most (red) and least (green) divergent within or between average distances.

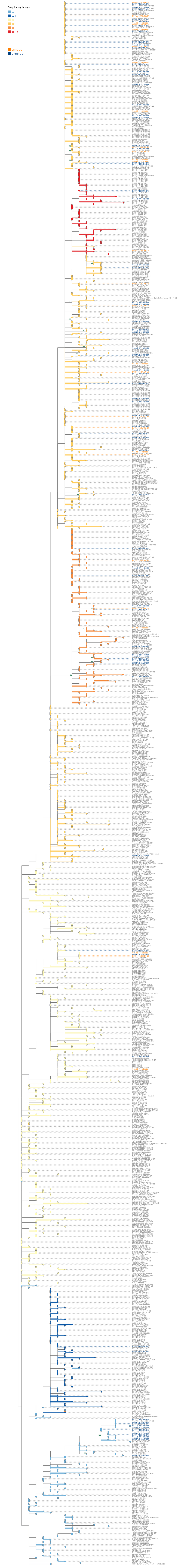

Supplemental Figure 6. Phylogenetic tree with all bootstrap values >65. Phylogenetic tree shown in Figures 3 and 4, but with bootstraps labeled on their corresponding nodes. Only bootstrap values greater than 65 are shown. Phylogenetic tree is colored as in Figure 4.

Supplemental Tables

Supplemental Table 1. Aggregate JHHS diagnostic tests by date. Number of positive tests within the Johns Hopkins Health System on each day during March 2020.

Supplemental Table 2. Aggregate sample metadata. Clinical metadata for all tested samples within the Johns Hopkins Health System, for all 143 samples sequenced in this study, and for the 114 patient samples that produced complete genomes. Results are grouped into appropriate bins to protect patient privacy.

Supplemental Table 3. Sequencing metrics. Sample names and accession numbers (where relevant) for all 143 samples selected for sequencing alongside sequencing metrics. Clade assignments using the Pangolin and NextStrain nomenclature systems are also provided for each sample that resulted in a whole genome sequence. One sample (MDHP-00146) was submitted to public repositories prior to enforcing a strict threshold of at least 27,000 unambiguous nucleotides, and therefore has an accession number but was not included in this analysis.

Supplemental Table 4. Single nucleotide polymorphisms present in JHHS sequences. Single nucleotide polymorphisms (with annotations) present in the 114 complete genomes generated in this study, alongside the number of samples containing each variant. Sites with IUPAC ambiguity codes due to mixed allele frequencies are also included.

Supplemental Table 5. Accession numbers of samples used in phylogenetic analysis. Accession numbers and submitting lab information for all 1279 sequences included in our phylogenetic tree.

Supplemental Table 6. Accession numbers of samples used in genetic distance analysis. Geographic group assignments for all sequences used in genetic distance analysis, listed by accession number. This table includes accession numbers for sequences included in the Global 3K dataset but were not included in the phylogenetic tree (which is based on the Global 1K dataset).

Supplemental Table 7. Quartile values for pairwise genetic distances within each geographic region. Mean, median, min, max, Q1, and Q3 values for the pairwise genetic distance distributions of each geographic group used in our analysis.
